# Complementary medical health services: a cross sectional descriptive analysis of a Canadian naturopathic teaching clinic

**DOI:** 10.1186/s12906-015-0550-6

**Published:** 2015-02-28

**Authors:** Deborah A Kennedy, Bob Bernhardt, Tara Snyder, Viviana Bancu, Kieran Cooley

**Affiliations:** Research Department, Canadian College of Naturopathic Medicine, Toronto, Canada; President’s Office, Canadian College of Naturopathic Medicine, Toronto, Canada; Clinic Administration, Canadian College of Naturopathic Medicine, Toronto, Canada; Information Services, Canadian College of Naturopathic Medicine, Toronto, Canada

**Keywords:** Complementary alternative medicine, CAM, Health care, Health professionals, Health services, Naturopathy, Ontario, Teaching clinic, Patient metrics

## Abstract

**Background:**

Historically, alongside regulatory and jurisdictional differences in scope of practices, practice patterns of naturopathic doctors (NDs) have varied widely to promote holistic or whole-person treatment using a variety of therapies including: controlled substances, minor surgery, a variety of complementary therapies, as well as both novel and conventional assessments. However, little is known about the observed practice patterns of NDs, the services provided to their patients, or the type of conditions for which patients of NDs are seeking treatment. In order to address this gap, a cross-sectional descriptive analysis of the largest Canadian teaching clinic for NDs was undertaken to better understand the services provided to the community and increase the knowledge regarding the use of naturopathic medicine.

**Methods:**

Data stemmed from two sources at the Toronto, Ontario clinic: a passive patient satisfaction survey, and the clinic’s point-of-sale (POS) system. Data included patient demographics, postal codes, health services utilization, ICD-10 codes, therapies employed, along with other data relating to the financial transactions associated with the visit. Simple descriptive statistics and the Kruskal-Wallis test were used to compare different age-based groups and examine health services use between years. This study was approved by the Research Ethics Board of the Canadian College of Naturopathic Medicine.

**Results:**

13,412 patients were treated in 76,386 patient visits spanning three clinic years. Median age of patients was 37; females outnumbered males (2.6:1) in all age-based groups except the pediatric population. In the patient satisfaction survey, there were 1552 potential survey respondents; with 118 responses received (response rate: 7.6%). Obtaining health education, health prevention and help with chronic health conditions were the primary motivators for patient visits identified in the patient survey.

**Conclusion:**

The clinic attracts people from a wide area in the metropolitan Toronto and surrounding region with health concerns and diagnoses that are consistent with primary care, providing health education and addressing acute and chronic health conditions. Further explorations into health services delivery from the broader naturopathic or other complementary/alternative medical professions would provide greater context to these findings and expand understanding of the patients and type of care being provided by these health professionals.

**Electronic supplementary material:**

The online version of this article (doi:10.1186/s12906-015-0550-6) contains supplementary material, which is available to authorized users.

## Background

In North America, the naturopathic profession is considered to be poised to be able to integrate conventional and CAM therapies and offer holistic primary care services supporting prevention and management of chronic health conditions [1-3]. Despite this bold statement, relatively little is known about the observed practices of regulated naturopathic doctors. Some research attests to effectiveness of the naturopathic approach, as measured by pragmatic, whole-practice randomized controlled trials [4-8], as well as observational studies [9-11] and patient experiences [12], while a much larger, and emerging evidence base exists for individual therapies for various conditions, some supportive of use, some demonstrating a lack of clear efficacy. It has also been posited that a substantial amount of value in naturopathic care is related to the quality time spent in patient care to achieve understanding and compliance, as well as the patient-centered collaborative model that naturopathic doctors bring to the patient-practitioner encounter [13]. This statement, as well as others which position the naturopathic profession as primary health care providers and experts in preventive health and chronic disease management [14] deserve further investigation and understanding, particularly given the eclectic and somewhat contentious nature of the naturopathic scope of practice and concerns over the changing regulatory climate in Canada [15-18].

Despite the myriad of clinical practices, some more evidence-based than others, it is clear that naturopathic doctors are delivering health care services, often as part of the private sector, engaging in culturally appropriate, patient-centered primary care delivery that may be filling gaps in underserviced areas or acting as part of an integrated model of patient care [19]. However, there is a dearth of literature fraught, historically, with significant challenges in the quest to examine the daily clinical reality of patients seen under naturopathic care in general [20]. Serving as a ‘living laboratory’, such information is vital for growth in research into patient-identified needs and treatment information (e.g., approaches to diagnosis, treatment decisions, impact on quality of life, and disease specific outcome measures) in naturopathic care. More germane to this cross-sectional study, comprehensive understanding of these components of overall care is essential to evaluation of the clinical environment as well as the effectiveness of prescribed treatments in order to inform the public, support interprofessionalism and collaboration, and provide a basis for targeting future research. Despite challenges and consequences identified in the exploration of health services research for complementary and alternative medical professions [21], there is merit in efforts to provide detailed descriptive data to understand the role that the naturopathic medicine may provide with respect to health care, particularly with respect to disease management, health promotion, prevention, chronic care management and patient education and empowerment [22].

In addition to dietary and lifestyle counseling, naturopathic doctors often prescribe a number of different complementary/alternative therapies including, but not limited to: acupuncture and Asian medicine, botanical (herbal) medicine, homeopathy, physical therapies (e.g. massage and manual bodywork, chiropractic manipulation and hydrotherapy), and natural health products (e.g. vitamins, minerals, amino acids, etc.) in oral, intramuscular and intravenous forms. Historically, and alongside regulatory and jurisdictional differences in scope of practices, practice patterns of naturopathic doctors have varied widely to include controlled substances (i.e. pharmaceutical drugs), minor surgery, meditation/visualization, Ayurvedic medicine, reflexology, as well as both novel (e.g. applied kinesiology, dark field microscopy), and conventional (i.e. laboratory testing) assessments [1,23]. Most recently, efforts to define academic competencies amongst the North American training institutions may be providing some normative influence on practice patterns of NDs, though it may be premature to speculate on or attempt to quantify these changes at this time [24].

The Canadian College of Naturopathic Medicine is Canada’s oldest and largest institute for naturopathic education and research and boasts one of the largest naturopathic teaching clinics in North America – the Robert Schad Naturopathic Clinic (RSNC). The clinic has been operating at its current location since the Fall of 1999, and hosts over 25,000 patient visits per year. Though no formal partnership exists, the clinic is geographically located in the North York region of Toronto, within the Central Local Health Integration Network (LHIN), one of Ontario’s 14 LHINs.

At the RSNC, student interns in their fourth and final year of study, provide patient care under the supervision of experienced, registered naturopathic doctors. The primary interaction is between patient and intern, however all treatment plans must be approved by the supervising naturopathic doctor. In addition to being a vibrant learning environment, this institutional setting also represents the largest collection of naturopaths and patients of naturopathic doctors in Canada, making it a prime location for high volume health services research.

Currently, the clinic hosts a number of specialty services, including focused clinical delivery for patients with fibromyalgia, sports/rehabilitative medicine, and adjunctive cancer care as well as a specialty shift to address the pediatric population. The clinic operates under a fee-for-service model including provisions to address the concerns of low-income or financially needy patients with reduced fees for visits. Patients pay out-of-pocket for services, though many receive third party reimbursement through workplace or personal health insurance coverage for naturopathic medicine.

To date, there has been no formal, comprehensive, published evaluation of the types of patients, health conditions seen, or health services provided by naturopathic doctors at this clinic, or any other in Canada. In order to address this gap in the health services literature, a cross-sectional descriptive analysis of the RSNC was undertaken spanning the three most recent years of operation, and a patient satisfaction survey was undertaken over a targeted time-span in 2011 to better understand the service being provided to the community.

## Methods

The data analyzed came from two primary sources. The major source is the clinic’s point-of-sale (POS) system which is used to collect a record of patient demographics, conditions seen (tracked through ICD-10 codes), labs requested and therapies employed, along with other data relating to the financial transactions associated with the visit. The patient survey is a self-reporting mechanism for gathering information on a variety of demographic and attitudinal data for an individual patient. The self-reporting associated with the patient survey leaves the data open to sampling bias. A comparison with the POS data allows a rough assessment as to the extent the survey data reflects the demographics of the patients who visit the clinic.

### Patient survey

In order to provide some insight into the socio-demographic parameters of the patients that attend the RSNC clinic, which are not collected by the POS, a relevant portion of a patient satisfaction survey conducted in 2011 is included. When a patient checked in for their appointment, they were handed a copy of the paper survey and asked to complete and return the survey in a collection box. Survey responses contained no identifying data; responses were considered to be anonymous. The survey captured socio-demographic data (age, gender, education, employment and income), visit related data (frequency of appointments, length of time as a patient at RSNC, waiting times prior to appointment), knowledge about the clinic (referral source, illness focused shift knowledge) and healthcare behaviors (reasons for coming to the clinic, impact of NM treatment on visits to family doctor, etc.). The participants were presented with each question and a series of possible answers from which they selected their response. Consent was implied when a patient returned the completed survey to the collection box. The participants were provided with the option to complete a separate ballot to be entered into a draw for two free visits to the RSNC as compensation for participating in the survey. A copy of the actual survey is provided in Additional file 1 which was developed through the collaborative efforts of the Marketing and Academic departments.

### Data source

The data was extracted from the POS (Microsoft Dynamics Retail Management System, Microsoft version 2.0 2007) used to capture both the financial sales transactions for patient visits, laboratory services and specialized supplies (e.g. botanical tinctures, iscador), as well as, therapies and procedures completed by the interns, as required to track their academic progress (e.g. prescriptions for botanical medicines, acupuncture, nutrition counseling, etc.). The data was extracted from the POS system in six separate files (2 files for each of the 3 years) with the date ranges corresponding to a clinic year (~May-May). The following data items were extracted from the database: patient salutation, patient name, age, gender, date of birth, account number, transaction number, time, transaction date, item lookup code, item description, quantity, intern name, intern number, supervising naturopathic doctor, cashier name, department code, transaction work order type, as well as five fields for ICD-10 codes and associated outcomes. The two files extracted for each clinic year contained transaction level data of transactions associated with a patient naturopathic consultation and transactions related to the patient experience that were not associated with a naturopathic consultation respectively. For example, if the patient received a B12 injection at the same time as he/she were at the clinic for a naturopathic consultation, the B12 injection transaction would be found in the first file; however, if the patient walked into the clinic for a B12 injection as part of a previously prescribed series of injections and did not have a naturopathic consultation visit, the B12 injection transaction would be found in the second file. Adjustment transactions for intern related activity is also included in the second file. The extracted data files were summarized and the transaction totals were cross-checked with the operational reports for these same periods as a quality control check.

The data were stored on a password protect secure server accessible by the Research department only. Only aggregated data were analyzed.

### Data methods

For each clinic year, the two extracted files were merged together into one Excel spreadsheet and all transactions representing solely academic transactions, were removed from the merged file. The pivot table function in MSExcel was used to tabulate the visit level information based on unique patient identifier number. To determine the number of unique patients seen each year, the transaction visits were copied to a separate spreadsheet and sorted according to account number and then duplicate account number records were removed. Patient groups were created largely on the basis of age, with one exception, the cancer-related diagnosis group. The Pediatrics group was defined as those individuals less than 18 years of age, while the Senior group was defined as those 65 and older. The Adult group was defined as those individuals between 18 and 64 years of age. For the Cancer-related diagnosis group, a separate data extraction was performed for patients with specific cancer related ICD-10 codes in any one of the five ICD-10 database fields. The account numbers in this data file were used to flag patients for the cancer-related diagnosis group. The ICD 10 codes that included are: C00 through C97, D00 through D09, D37 through D48, Y43.3, and Z08.2.

### Determination of top health concerns by patient group

Clinic interns are responsible for identifying the most appropriate ICD-10 codes to describe their patients’ health concerns. This data is captured by the POS system. Since patient visits do span multiple clinic years, all patient visit transactions were initially consolidated into one MSExcel spreadsheet. From here, separate visit spreadsheets were created for each patient group (pediatrics, adults, seniors, cancer-related diagnosis). For each patient group visit spreadsheet the initial patient visit ICD-10 codes were totaled and the appropriate description and group associated with each code. Totals were obtained by group and the top 10 groups were reported.

### Determination of treatment modality

Clinic interns indicate which treatment(s) have been provided to patients at each visit, for example: prescriptions for homeopathy, botanical tinctures, natural health products or acupuncture treatment, by using specific codes. For the purpose of determining the proportion of patients that received each one of these treatment modalities, the patient visit transactions with the code corresponding to these treatments were placed into a spreadsheet and duplicated entries removed.

### Informed consent

All patients of RSNC receive an information letter regarding data collection for research and teaching purposes and provide informed consent prior to treatment. This study was approved by the Research Ethics Board of the Canadian College of Naturopathic Medicine.

### Statistical analysis

The Kruskal Wallis test was used to compare the number of patients in each of the different patient groups across the three years. A significance level of p < 0.05 was established. Stats Direct version 2.7.8 (Cheshire, UK) was used to perform the statistical analysis.

## Results

### Patient survey

The patient satisfaction survey was available from March 10 to April 10, 2011. During this time, there were 1,552 patients that were seen at the clinic and 118 surveys completed, representing a response rate of 7.6%. The socio-demographic characteristics of the survey participants are summarized in Additional file 2. The majority of the respondents were between the ages of 18–39 years, female with an income of less than $40,000 per year, employed with a university education and had been patients at the RSNC for more than one year.

The primary reasons indicated in the patient survey for attending the RSNC included; obtaining health education, health prevention and for help with chronic health conditions. Most survey respondents indicated that they felt that they saw their family doctor less than they did before coming to the clinic and 75% said that they used the clinic for most of their health needs (see Additional file 3).

### Patient and visit data summary

There were 13,412 people who were seen as patients at the RSNC between May 9, 2010 and May 5, 2013. Table 1 summarizes the number of patients seen yearly, by age group and gender. The patient population grew by 2.8% in 2011 and by almost 11% in 2012. The pediatric population is a growing segment of the patient base. As a teaching clinic the cost-per-visit is considerably below that of naturopathic doctors in the surrounding community; however, the RSNC offers a reduced visit rate to those individuals in financial need. There was an average of 4.2% of the patients in each year in this category, with approximately 6% of the visits occurring at a reduced rate in each year. There were no statistical differences (Kruskal Wallis p = 0.94) found in the distribution of the patient groups across the three clinic years in terms of both the patient population mix (i.e., predefined patient groups) and the patient population visit mix (data not shown). There were a total of 76,386 patient visits across the three clinic years included in this report. The annual breakdown is summarized in Table 1. There was a 12.6% increase in the number of visits between the clinic year 2010 and the end of clinic year 2012. In the clinic year 2012, for all patients, the mean number of visits was 5.6 visits (SD: 6.2 visits) (data not shown).

**Table 1:** **Number and distribution of patients and visits by clinic year**

**Clinic year**	**2010**		**2011**		**2012**	
**Patients**	**N**		**N**	**% change**	**N**	**% change**
Total	4233	-	4352	2.8	4827	10.9
Visits	24087	-	25172	4.5	27127	7.8
**Age breakdown**	**N**	**(%)**	**N**	**(%)**	**N**	**(%)**
Peds (≤17)	341	(8.1)	401	(9.2)	465	(9.6)
Adults (18–64)	3332	(78.7)	3389	(77.9)	3779	(78.2)
Seniors (65+)	506	(11.9)	533	(12.2)	562	(11.6)
Unknown	54	(1.3)	29	(0.7)	21	(0.4)
**Gender- patients**	**N**	**(%)**	**N**	**(%)**	**N**	**(%)**
Male	886	(26.0)	180	(27.3)	1315	(27.6)
Female	2519	(73.9)	3122	(72.4)	3443	(72.3)
Unknown	5	(0.1)	11	(0.3)	3	(0.1)
No data	823		39		66	

**Figure 1:**
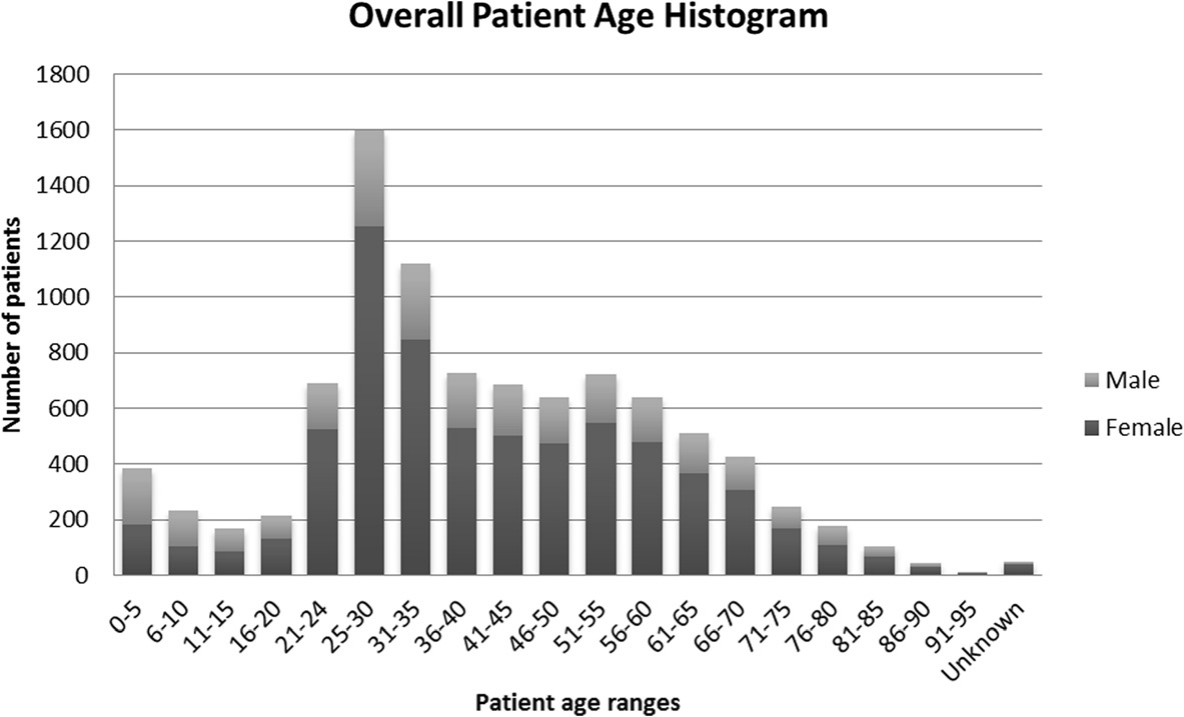
**Distribution of patient ages seen at Robert Schad Naturopathic Clinic by gender, 2010–2012.**

Figure 1 provides an overview of the age distribution of the patients that were seen at the RSNC over the 3 year period. The median age is 37 years and the average patient is 40 years of age. The majority of the patients are between 18–64 years, with the senior population slightly more represented than the pediatric population; however, the pediatric population has been slowly growing over the three year period. Female patients between the ages of 25–30 years comprise the largest segment of the patient population (Figure 1) and women outnumber men at a ratio of 2.6:1. An exception to the predominance of female patients is in the ≤10 age group, where males were seen slightly more that female children (Figure 1). The ratio of male to female pediatric patients is 1.04:1.

**Figure 2:**
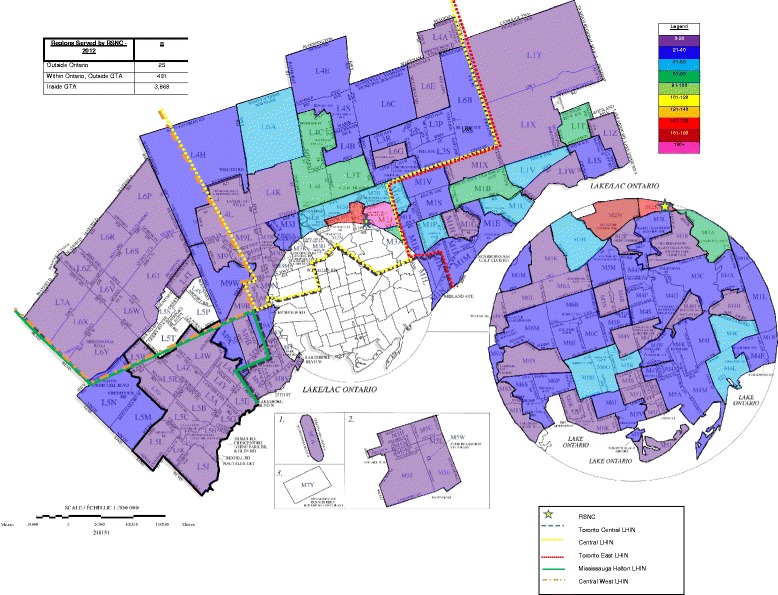
**Robert Schad Naturopathic Clinic patient density map.**

Figure 2 shows the geographical distribution of the patients based on postal code associated with the home addresses of the patients. The greatest patient density is just around the location of the teaching clinic. The next largest area of patient density (green) is located north of the clinic location and above the boundary of the City of Toronto (Steeles Avenue). The areas of the greatest patient density are located north and east of the clinic. There are some small pockets of patient density south of the clinic location. The clinic serves not only the Greater Toronto Area (GTA) but also attracts some patients from outside this area and outside of the province of Ontario. Patients, for example, may be visiting family in the GTA for an extended period and chose to come to the clinic for assistance with their health. Since this map does represent the home addresses of the patients, rather than their business address, it is possible that patients’ would choose the clinic because of its proximity to a work location and seek appointments either on the lunch hour or after work.

### Reasons for visits and associated treatments

The top health concerns in each patient group are summarized (see Additional file 4). The reasons for seeking naturopathic assistance are varied, both within and across the patient groups. Nasal and sinus conditions are represented in each of the patient groups as part of the top 10. Anxiety, as the top concern in the Adult patient group, could be confounded by the proximity of the teaching clinic to the naturopathic school. Many of the students’ could access the services of the clinic to assist with the anxiety and stresses of student life. Menstrual disorders as the second most frequently cited health concern may well be associated with a higher proportion of adult patients that are female. Joint, sleep and thyroid disorders are among the top 10 health concerns for both the adult and the senior patient groups. Many of these health concerns are chronic in nature; however, patients do come to the RSNC for screening and other diagnostic laboratory assessments. Table 2 summarizes the number of screening and laboratory tests performed each year.

**Table 2:** **Number of select laboratory tests performed by clinic year**

**Clinic year**	**2010**	**2011**	**2012**
B12 (1 ml B12)	1183	1061	1013
B12 high dose (5 ml B12)	204	637	993
Rapid strep test	18	16	12
Liquid based pap smear	126	139	92
Vitamin D [25(OH)D_3_]	20	54	74
Serum ferritin	173	271	211
Hemoglobin A1c	46	41	42
Thyroid stimulating hormone	54	81	72
Swab (general)	68	83	83
Skin (KOH and culture)	41	33	36
Glucometer	4	12	3
Complete blood count	200	233	166

**Figure 3:**
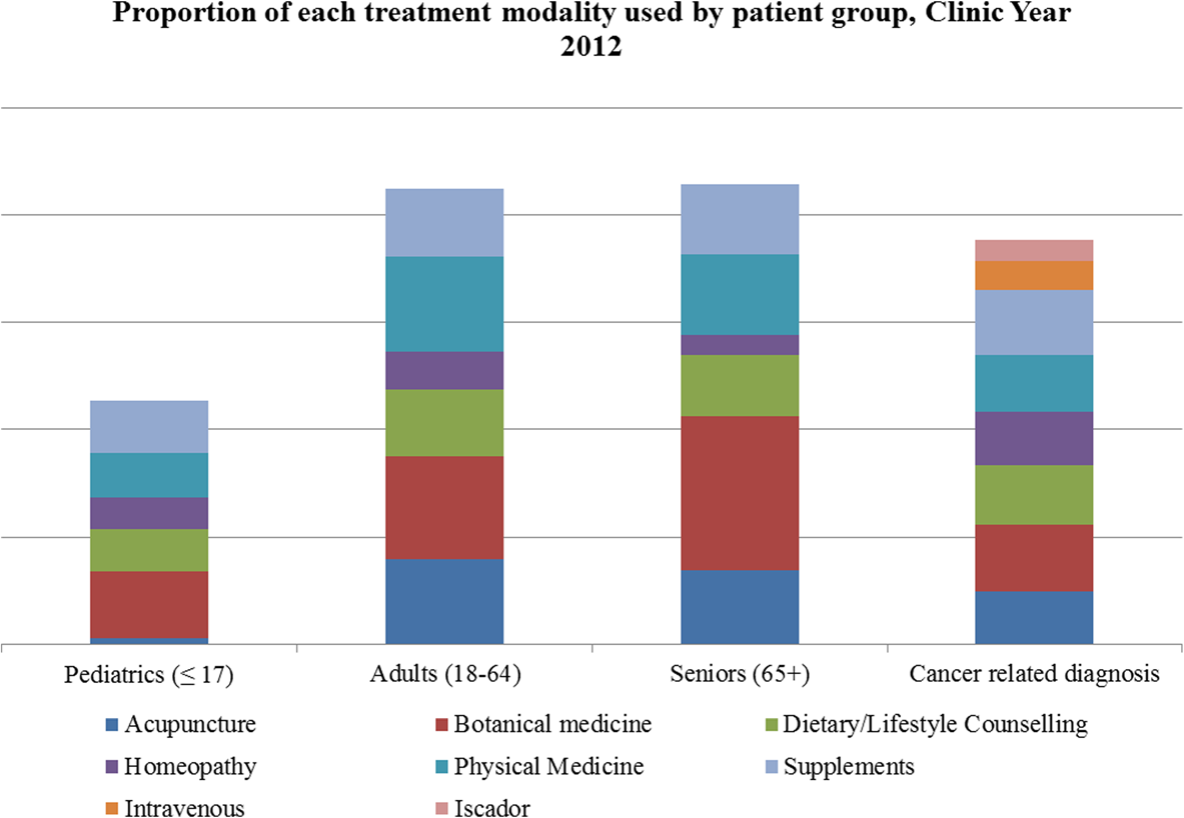
**Proportion of each treatment modality used, by patient group for clinic year 2012.**

Patients at the clinic can receive a number of different therapies to address their health concerns. Figure 3 graphically illustrates the proportion of patients that received acupuncture, botanical medicine, dietary/lifestyle counseling, homeopathy, physical medicine (bodywork, castor oil packs, hydrotherapy, manipulation, and peat baths) and supplements as components of their treatment protocol. The use of these treatment modalities in each patient group is not mutually exclusive. Botanical medicines are the most frequently used treatment modality across all of the patient groups. In both the Adult and Senior groups, the prevalence of the treatment modalities is botanical medicines, acupuncture, physical medicine and then supplements. In the cancer-related diagnosis group, the most frequently used modality is botanical medicine, followed by supplements, and dietary/lifestyle counseling. Acupuncture and homeopathy are used with equal frequency in this patient group. Not surprisingly, acupuncture is used in only a very small number of pediatric patients, with botanical medicine, supplements, and physical medicine as the top three modalities followed by homeopathy.

## Discussion

On average over 4,400 patients are seen every year at the RSNC. The gender and age of the patient population is representative of what has been identified in other CAM surveys, predominately female and between the ages of 30 and 40 [25-30]. The patient demographic profile is similar to that reported by Chamberlain et al. in their report of the consolidated naturopathic medicine teaching clinic patient profile [31]. Though, the RSNC sees a slightly older patient demographic. In a previously conducted chart review study of the RSNC, the gender mix on the pediatric population was a 1.09:1 ratio of female: male [32]. While in this analysis the ratio is 1:1.04, with male children slightly exceeding female, this differs from the Adult and Senior groups where female patients outnumber the male patients, 2 to 1. Reasons for this gender-based difference between these age groups have yet to be fully explored.

Hawk et al. in their survey of CAM practitioners reported that in a 12 month period, patients consulting with naturopathic doctors were seen most frequently for between 2 to 5 visits per patient [33]. The patient population at the RSNC is seen just slightly more than the Hawk results suggested. This may perhaps be expected in light of the teaching nature of the RSNC clinic environment.

Williams et al. in their geographic analysis of alternative health care consultations in Canada found that geography was not a significant factor in determining consultations [34]. This would appear to be born out, to a certain extent by the geographical area served by the RSNC. The western boundary of the map represents a distance of 55 kilometers (km) (approximately 34 miles) from the clinic location, the northern boundary 32 km (approximately 20 miles) and the eastern boundary 36 km (approximately 22 miles), with the heaviest density in the north and east, a total of 3,276 square kms (1,232 square miles). People are driving considerable distance to attend the clinic. Of the 565 naturopathic doctors that maintain a primary practice location in the area represented by the map, 72.4% (409/565) are located in the City of Toronto, while 9.2% (52/565) are located in Mississauga [35]. The availability of other naturopathic clinics may be a factor to consider with respect to the geographic distribution of patients and could be a possible explanation for the lower patient density in these cities. Previous health geography assessments of total health supply in Ontario indicate a ‘cluster’ of complementary/alternative medical practitioners in relation to urban density measures, as well as indicators of community well-being [36].

The reasons for naturopathic visits of the pediatric population have shifted since the previous study conducted of the pediatric population of the RSNC. In 2002, Wilson et al. conducted a chart review of the pediatric patients attending the clinic and summarized their findings [32]. At that time, skin and gastrointestinal disorders were the most frequently cited visit reasons. The reason for pediatric patient visits still includes both skin and gastrointestinal disorders; however, periodic health assessment/screening is now the most frequent reason for pediatric visits. In Leung and Verhoef’s survey of parents, they found that 35.8% of the parents considered the ND to be the main healthcare provider for their child and 34.7% said that both the ND and MD were viewed equally as primary healthcare providers [37]. The rise of periodic health assessment/screening as one of the top reasons for visits in the pediatric population may be a trend.

A study on older adults’ (55+) use of naturopathic medical services in the Seattle, Washington area reported that top reasons for seeking care were fatigue, anxiety, diabetes, diarrhea and upper respiratory infections. There is little similarity between health concerns of the Washington Seniors and the RSNC Seniors groups, with diabetes the only common health concern. Some of the Washington Seniors’ health concerns could be characterized as acute in nature (diarrhea, upper respiratory infection (URI)), while the RSNC Seniors health concerns are more chronic and aligned with those chronic health conditions of concern in the aging Canadian population [38]. Since, the age ranges between the two studies are not perfectly aligned, the Washington study includes adults between 55 – 64 years, this may account for the difference between the health concerns of these two groups, though it is acknowledged that health approaches between East and West coast people are different [39].

The cancer conditions reported in the health concerns of those with a cancer-related diagnosis mirror those that also represent the top four newly diagnosed cancers in Ontario/Canada [40]. This suggests that people with cancer are seeking adjuvant support for their health concerns, rather than seeking primary treatment for a chronic cancer condition, chronic myeloid leukemia, as an example.

Statistic Canada’s Health Profile for the Toronto Health Unit data was included as the first column of Table 2. This data summarizes the percentage of the population in the region that suffers from the various health conditions listed [41]. A comparison between the health conditions seen at the RSNC in the adult population and the Toronto region demonstrates some overlap in health conditions.

The response rate of the patient satisfaction survey is low. There are a number of possible explanations. The most likely explanation is that the patients were provided the survey when they checked in for their appointment and may not have had any time to complete the survey prior to the start of their appointment. Rather than taking it with them to complete and return upon their next visit, they simply forgot about the survey. The survey was paper based and with the largest patient age demographic in the 25–35 age range and their corresponding comfort with smart phones, an electronic survey may be more appealing and have yielded a greater response rate. There is potential for selection bias in the patient satisfaction survey. The survey was open for one month only, which may have been insufficient to capture the largest cross section of the patient population, since patients may only come 4–5 times per year.

### Limitations

The low response rate alongside a self-selecting sample of respondents is a limitation in the interpretation of findings from the patient satisfaction survey and is one of the limitations of this study. This data can only provide limited insight into the resulting health behaviors of people who attend the teaching clinic. The source of the data is from a retail POS system, as such, there are limitations in both type and amount of data that can be captured and reported. Clinic interns, in consultation with their supervising naturopathic doctor, are responsible for assigning the ICD-10 diagnostic codes relevant to the patient. The assigned codes may only reflect the presenting health concerns of the patient, which may not necessarily be the focus of the treatment protocol. Further, the incompatible nature of the data from the patient satisfaction survey and the retail POS system prevents the cross-tabulation of the data to draw associations between patient health concerns and outcomes, and their satisfaction with the clinic. The data presented here is based on the activity of a naturopathic teaching clinic and therefore does not necessarily reflect the practice patterns of naturopathic doctors in private practice, who might serve a limited geographic area or scope of health concerns.

## Conclusion

This is the first report of the patient profile, top health care reasons for visits and treatments provided to patients that are seen at the largest naturopathic teaching clinic in North America. The clinic attracts people from a wide area in the metropolitan Toronto and surrounding region with health concerns and diagnoses that are consistent with primary care, including patients with acute, chronic and complex multi-morbid conditions. Patients to the clinic indicate that their reasons for coming include health education, health prevention/screening and help for their chronic health conditions. Further explorations into health services delivery from the broader naturopathic or other complementary/alternative medical professions would provide greater context to these findings and expand understanding of the patients and type of care being provided by these health care providers.

## Additional files

Additional file 1:
**Robert Schad naturopathic clinic patient satisfaction survey.**
Additional file 2:
**Socio-demographics of participants that completed the patient satisfaction survey.**
Additional file 3:
**Patient Survey: Health behavior results.**
Additional file 4:
**Top 10 health concerns, by patient group all clinic years.**

## Abbreviations

B12: Vitamin B12
CAM: Complementary and alternative medicine
ICD-10: International classification of diseases, version 10
GTA: Greater Toronto area
LHIN: Local health integration network
Km: Kilometer
ND: Naturopathic doctor
NM: Naturopathic medicine
POS: Point of sale
URI: Upper respiratory infection
