# Supplementary material for: Complementary medical health services: a cross sectional descriptive analysis of a Canadian naturopathic teaching clinic
Source: BMC Complement Altern Med. 2015 Feb 28;15:37. doi: 10.1186/s12906-015-0550-6 (PMC4362820; doi:10.1186/s12906-015-0550-6)
Supplement: Additional file 1: — Robert Schad naturopathic clinic patient satisfaction survey. [file 12906_2015_550_MOESM1_ESM.doc]

At the Robert Schad Naturopathic Clinic, your health and wellness, needs and expectations are important to us. Please help us serve you better by answering the following questions. Your responses are confidential and will in no way identify you. This survey will take approximately five minutes to complete.

**Demographic Information**

Age:

| - Under 18 | - 50 - 59 |
| --- | --- |
| - 18 - 29 | - 60 – 69 |
| - 30 - 39 | - 70 - 79 |
| - 40 - 49 | - Over 80 |

**Gender:**

- Female
- Male

Marital status:

| - Single | - Divorced/separated |
| --- | --- |
| - Married/common-law | - Widowed |

Annual household income:

| - Under $20,000 | - $61,000 - $80,000 |
| --- | --- |
| - $21,000 - $40,000 | - $81,000 - $100,000 |
| - $41,000 - $60,000 | - More than $100,000 |

Employment status:

| - Employed full-time (incl. self-employed) | - Non-CCNM student |
| --- | --- |
| - Employed part-time | - Unemployed |
| - CCNM student | - Retired |

What is your occupation? _______________________

Highest level of education completed:

| - University degree (including post-grad) | - Secondary school diploma |
| --- | --- |
| - College diploma | - Other |

**Country of origin**: ____________________________

**First language**: ______________________________

**Please provide the first three digits of your postal code:** ________________________________

**Your Clinic Experience:**

How many times have you visited RSNC?

- First visit
- 2 – 3 visits
- 4 – 10 visits
- 11 – 20 visits
- more than 20 visits

How long have you been a patient at RSNC?

- First visit
- Less than 1 month
- 1 – 6 months
- 7 months – 1 year
- More than one year

At what point in the day do you usually visit the RSNC?

- Morning
- Afternoon
- Evening
- Saturday morning
- Saturday afternoon

How long after your scheduled appointment time did you/do you usually wait in the waiting room?

- Less than 5 minutes
- 5 - 10 minutes
- 11 – 15 minutes
- 16 – 20 minutes
- Longer than 20 minutes

How did you hear about RSNC? (Check all that apply)

| - TTC advertising | - Newsletter delivery to residence |
| --- | --- |
| - Media article/TV | - Radio advertising |
| - Company health and wellness event | - CCNM Open House |
| - CCNM student | - CCNM staff/faculty |
| - Newspaper advertising | - Friend/non-CCNM |
| - Family/non-CCNM | - RSNC patient |
| - Satellite clinic patient | - Word of mouth |

We’re always looking for new ways to promote the RSNC in our community. If you have any suggestions as to where we could advertise the services provided at the clinic, please let us know.

____________________________________________________________________________________________________________________________________________________________________________________

Are you aware of the following focused shifts and programs?

- Pediatrics
- Sports medicine
- Adjunctive cancer care
- Be Your Best Self & Be Your Best Self Kids & Teens

Are your RSNC visits covered by your extended health plan?

- Yes
- No

What reason(s) do you come to the RSNC to receive treatment? (check all that apply)

❑ Location

❑ Ongoing care (I’ve been a patient here for at least 2 years)

❑ Reduced price

❑ Referred by friend/relative

❑ Referred by my family doctor

❑ Referred by other health-care practitioner

❑ Supporting a 4^th^ year student

❑ Trust ND to be part of care I receive for my health

❑ Trust ND to be my primary health care provider

❑ Other(s) _________________________________________________________________________

_______________________________________________________________________________

Please check which health-care provider you would primarily visit to receive care for the following conditions: (if not applicable, please put N/A in ‘Other’)

Health screening ❑ ND ❑ Family doctor/GP ❑ Other ______________

Diagnosis of your medical concerns ❑ ND ❑ Family doctor/GP ❑ Other ______________

Acute conditions (e.g., colds/flus) ❑ ND ❑ Family doctor/GP ❑ Other ______________

Rehabilitation from injury ❑ ND ❑ Family doctor/GP ❑ Other ______________

Chronic condition(s) ❑ ND ❑ Family doctor/GP ❑ Other ______________

Mental health issues ❑ ND ❑ Family doctor/GP ❑ Other ______________

Health prevention/health screening ❑ ND ❑ Family doctor/GP ❑ Other ______________

Health education ❑ ND ❑ Family doctor/GP ❑ Other ______________

Vaccination/vaccine education ❑ ND ❑ Family doctor/GP ❑ Other ______________

2nd opinion on my health ❑ ND ❑ Family doctor/GP ❑ Other ______________

Managing my overall care ❑ ND ❑ Family doctor/GP ❑ Other ______________

For any of the above, if you would **prefer** to primarily go to a health-care provider **other** than the one you checked, please tell us why___________________________________________________________________

__________________________________________________________________________________________

Since coming to the RSNC, I see my family doctor:

❑ More frequently

❑ About the same amount

❑ Less frequently

❑ Not applicable

Please tell us why_______________________________________________________________________

______________________________________________________________________________________

Do you come to RSNC for most of your health needs? Yes or No

If no, what are the top 3 reasons why you do not see your RSNC intern for more of your health care needs (check all that apply):

❑ It is too expensive

❑ It is inconvenient

❑ Lack of continuity (my intern leaves at the end of the each school year)

❑ I would prefer to see a more experienced ND for some of my health needs

❑ I receive adequate care elsewhere

❑ I would prefer more collaborative care

❑ Other:______________________________________________________________________________

How did you travel here today?

| - Drove myself | - Someone drove me here |
| --- | --- |
| - TTC (subway only) | - TTC (subway and/or streetcar/bus) |
| - Walked | - Biked |

How much travel time was involved (one way)?

| - Less than 15 minutes | - 15 – 30 minutes |
| --- | --- |
| - 30 – 45 minutes | - More than 1 hour |

Did you visit the RSNC website before your first clinic visit?

- Yes
- No

If yes, how many times have you visited the RSNC website?

- Once
- 1 – 5 times
- More than 5 times

Did you find the information you required on the website?

- Yes
- No

If not, what might we add? ___________________________________________________________________

If you are a new patient (within the last 6 months), were you made aware of the new patient brochure (either by mail or directed to our website?)

- Yes
- No

**Your Clinic Visit:**

Please indicate your level of agreement with each of the following statements.

The clinic: Strongly Strongly

Disagree Agree

| Was easy to find | 1 | 2 | 3 | 4 | 5 | 6 | 7 |
| --- | --- | --- | --- | --- | --- | --- | --- |
| Was clean, tidy and reflected a professional environment | 1 | 2 | 3 | 4 | 5 | 6 | 7 |
| Has operating hours that are convenient for me | 1 | 2 | 3 | 4 | 5 | 6 | 7 |
| The treatment rooms met my needs | 1 | 2 | 3 | 4 | 5 | 6 | 7 |

Additional comments:

The booking and reception staff: Strongly Strongly

Disagree Agree

| Made it easy for me to book or change my appointment | 1 | 2 | 3 | 4 | 5 | 6 | 7 |
| --- | --- | --- | --- | --- | --- | --- | --- |
| Were professional and courteous | 1 | 2 | 3 | 4 | 4 | 6 | 7 |
| Adequately prepared me for my first visit | 1 | 2 | 3 | 4 | 5 | 6 | 7 |
| Answered my questions | 1 | 2 | 3 | 4 | 5 | 6 | 7 |
| Returned my phone calls within a reasonable time | 1 | 2 | 3 | 4 | 5 | 6 | 7 |
| Made me feel welcome when I arrived at the clinic | 1 | 2 | 3 | 4 | 5 | 6 | 7 |
| Asked me if I wanted to book another appointment | 1 | 2 | 3 | 4 | 5 | 6 | 7 |

My intern: Strongly Strongly

Disagree Agree

| Took enough time with me | 1 | 2 | 3 | 4 | 5 | 6 | 7 |
| --- | --- | --- | --- | --- | --- | --- | --- |
| Addressed my concerns | 1 | 2 | 3 | 4 | 5 | 6 | 7 |
| Appeared competent and confident | 1 | 2 | 3 | 4 | 5 | 6 | 7 |
| Appeared prepared for my appointment | 1 | 2 | 3 | 4 | 5 | 6 | 7 |
| Was dressed professionally | 1 | 2 | 3 | 4 | 5 | 6 | 7 |
| Explained my treatment plan to my understanding including timelines | 1 | 2 | 3 | 4 | 5 | 6 | 7 |
| Explained where to purchase supplements | 1 | 2 | 3 | 4 | 5 | 6 | 7 |
| Helped me feel comfortable and relaxed | 1 | 2 | 3 | 4 | 5 | 6 | 7 |
| Provided clear written instructions on how to take my supplements and tinctures | 1 | 2 | 3 | 4 | 5 | 6 | 7 |
| Suggested when I should return for my next visit | 1 | 2 | 3 | 4 | 5 | 6 | 7 |

The supervising ND: Strongly Strongly

Disagree Agree

| Played an active role in managing the case | 1 | 2 | 3 | 4 | 5 | 6 | 7 |
| --- | --- | --- | --- | --- | --- | --- | --- |
| Spent enough time in the room with the intern and me | 1 | 2 | 3 | 4 | 5 | 6 | 7 |
| Appeared to have a good working relationship with my intern | 1 | 2 | 3 | 4 | 5 | 6 | 7 |
| Added value to my clinic experience | 1 | 2 | 3 | 4 | 5 | 6 | 7 |
| Was dressed professionally | 1 | 2 | 3 | 4 | 5 | 6 | 7 |
| Was easy to contact between appointments | 1 | 2 | 3 | 4 | 5 | 6 | 7 |

I spent too much time: Strongly Strongly

Disagree Agree

| In the waiting room before my treatment | 1 | 2 | 3 | 4 | 5 | 6 | 7 |
| --- | --- | --- | --- | --- | --- | --- | --- |
| Waiting for my supervising ND to attend a treatment/discuss my case | 1 | 2 | 3 | 4 | 5 | 6 | 7 |
| Waiting for lab services | 1 | 2 | 3 | 4 | 5 | 6 | 7 |
| Waiting to pay | 1 | 2 | 3 | 4 | 5 | 6 | 7 |

(If you did not use laboratory services or testing, please proceed to the following question):

Laboratory services: Strongly Strongly

Disagree Agree

| Were provided in a professional manner | 1 | 2 | 3 | 4 | 5 | 6 | 7 |
| --- | --- | --- | --- | --- | --- | --- | --- |
| Were provided in a timely fashion | 1 | 2 | 3 | 4 | 5 | 6 | 7 |
| Allowed me to feel comfortable during lab procedures | 1 | 2 | 3 | 4 | 5 | 6 | 7 |

Overall: Strongly Strongly

Disagree Agree

| Naturopathic medicine plays an important role in my overall health and well-being. | 1 | 2 | 3 | 4 | 5 | 6 | 7 |
| --- | --- | --- | --- | --- | --- | --- | --- |
| RSNC plays an important role in my overall health and well-being. | 1 | 2 | 3 | 4 | 5 | 6 | 7 |
| RSNC provides good value for my money. | 1 | 2 | 3 | 4 | 5 | 6 | 7 |
| I would refer/have referred friends and family to the RSNC. | 1 | 2 | 3 | 4 | 5 | 6 | 7 |
| My billing was comprehensive and accurately reflected the services rendered. | 1 | 2 | 3 | 4 | 5 | 6 | 7 |

In your opinion, how can we improve the Robert Schad Naturopathic Clinic?

____________________________________________________________________________________________________________________________________________________________________________________

**Thank you for your feedback. Your input will help us provide better service to our patients.**

**INCLUDE YOUR NAME AND CONTACT INFORMATION ONLY IF YOU WISH TO BE ENTERED INTO OUR DRAW FOR RSNC GIFT CERTIFICATES:**

**Name (please print): ____________________________________________________________________**

**Daytime contact information:**

**Telephone: _____________________________________________________**

**E-mail: _________________________________________________________**
