# Supplementary material for: Complementary medical health services: a cross sectional descriptive analysis of a Canadian naturopathic teaching clinic
Source: BMC Complement Altern Med. 2015 Feb 28;15:37. doi: 10.1186/s12906-015-0550-6 (PMC4362820; doi:10.1186/s12906-015-0550-6)
Supplement: Additional file 2: — Socio-demographics of participants that completed the patient satisfaction survey. [file 12906_2015_550_MOESM2_ESM.docx]

**Additional File 2:** Socio-demographics of participants that completed the patient satisfaction survey.

| **Age** | **N** | | | **%** | |
| --- | --- | --- | --- | --- | --- |
| 18 - 29 | 30 | | | 25.4 | |
| 30 - 39 | 29 | | | 24.6 | |
| 40 - 49 | 22 | | | 18.6 | |
| 50 - 59 | 21 | | | 17.8 | |
| 60 - 79 | 14 | | | 13.6 | |
| **Gender** |  | | |  | |
| Female | 96 | | | 83.5 | |
| Male | 19 | | | 16.5 | |
| **Marital status** |  | | |  | |
| Single | 54 | | | 46.2 | |
| Married/common-law | 50 | | | 42.7 | |
| Divorced/separated/widowed | 13 | | | 11.1 | |
| **Annual household income** |  | | |  | |
| Under $20,000 | 29 | | | 26.1 | |
| $21,000 - $40,000 | 19 | | | 17.1 | |
| $41,000 - $60,000 | 20 | | | 18.0 | |
| $61,000 - $80,000 | 15 | | | 13.5 | |
| $81,000 - $100,000 | 17 | | | 15.3 | |
| More than $100,000 | 11 | | | 9.9 | |
| **Employment status** |  | | |  | |
| Employed full time (incl. self-employed) | 55 | | | 47.4 | |
| Employed part time | 20 | | | 17.2 | |
| Student | 23 | | | 19.8 | |
| Unemployed | 9 | | | 7.8 | |
| Retired | 9 | | | 7.8 | |
| **Highest level of education completed** | | | | | |
| University degree (including post-grad) | 65 | | | 56.0 | |
| College diploma | 34 | | | 29.3 | |
| Secondary school diploma | 13 | | | 11.2 | |
| Other | 4 | | | 3.4 | |
| **How many times have you visited RSNC?** | | | | | |
| First visit | 9 | | 7.8 | | |
| 2 - 3 visits | 7 | | 6.1 | | |
| 4 - 10 visits | 35 | | 30.4 | | |
| 11 - 20 visits | 14 | | 12.2 | | |
| more than 20 visits | 50 | | 43.5 | | |
| **How long have you been a patient at RSNC?** | **N** | **%** | | | |
| First visit | 9 | | | | 7.9 |
| Less than 1 month | 2 | | | | 1.8 |
| 1 - 6 months | 39 | | | | 34.2 |
| 7 months - 1 year | 15 | | | | 13.2 |
| More than 1 year | 49 | | | | 43.0 |
