# Supplementary material for: Complementary medical health services: a cross sectional descriptive analysis of a Canadian naturopathic teaching clinic
Source: BMC Complement Altern Med. 2015 Feb 28;15:37. doi: 10.1186/s12906-015-0550-6 (PMC4362820; doi:10.1186/s12906-015-0550-6)
Supplement: Additional file 3: — Patient Survey: Health behavior results. [file 12906_2015_550_MOESM3_ESM.docx]

**Additional File 3: Patient Survey: Health behavior results**

| **Since coming to the RSNC, I see my family doctor:** | **N** | **%** |  |  |
| --- | --- | --- | --- | --- |
| More frequently | 5 | 4.5 |  |  |
| About the same amount | 36 | 32.4 |  |  |
| Less frequently | 61 | 55.0 |  |  |
| Not applicable | 9 | 8.1 |  |  |
|  |  |  |  |  |
| **Do you come to RSNC for most of your health needs?** | **N** | **%** |  |  |
| Yes | 79 | 75.2 |  |  |
| No | 26 | 24.8 |  |  |
|  |  |  |  |  |
| **Please check which health-care provider you would primarily visit to receive care for the following conditions:** | **N** | **ND**  **%** | **Other***  **%** | **ND & Other**  **%** |
| Health education | 104 | 71.8 | 13.6 | 14.6 |
| Health prevention | 104 | 63.5 | 16.3 | 20.2 |
| Chronic condition(s) | 103 | 61.8 | 16.7 | 21.6 |
| Second opinion on my health | 105 | 62.1 | 18.4 | 19.4 |
| Managing my overall care | 108 | 61.9 | 12.4 | 25.7 |
| Mental health issues | 98 | 47.3 | 33.3 | 19.4 |
| Rehabilitation from injury | 86 | 44.7 | 40.4 | 14.9 |
| Acute conditions (e.g., colds/flus) | 98 | 41.2 | 39.2 | 19.6 |
| Health screening | 106 | 28.3 | 48.1 | 23.6 |
| Diagnosis of your medical conditions | 107 | 20.6 | 52.3 | 27.1 |
| Vaccination/vaccine education | 90 | 19.1 | 68.5 | 12.4 |

*This includes Family doctor/General Practitioner and other healthcare providers.
