# Supplementary material for: Complementary medical health services: a cross sectional descriptive analysis of a Canadian naturopathic teaching clinic
Source: BMC Complement Altern Med. 2015 Feb 28;15:37. doi: 10.1186/s12906-015-0550-6 (PMC4362820; doi:10.1186/s12906-015-0550-6)
Supplement: Additional file 4: — Top 10 health concerns, by patient group all clinic years. [file 12906_2015_550_MOESM4_ESM.docx]

**Additional File 4~~Table 2~~:** Top 10 health concerns, by patient group all clinic years.

|  | **Robert Schad Naturopathic Medicine Clinic** | | | |
| --- | --- | --- | --- | --- |
| **Regional Health Conditions^41^**  **(% of population)** | **Pediatrics (≤ 17)** | **Adults (18-64)** | **Seniors (65+)** | **Cancer–related diagnosis** |
| - Overweight or obese (44.8) - High blood pressure (15.9) - Pain prevents activities (15.3) - Arthritis (14.0) - Mood disorder (7.1) - Diabetes (5.3) - Asthma (5.9) - COPD (1.9) | - Health Assessment - Nasal & Sinus Congestion - Atopic Dermatitis - Ear Symptoms - Dermatitis - Cough - URTI - Constipation - Asthma - Hyperkinetic Disorders | - Anxiety - Menstrual Disorders - Malaise & fatigue - Nasal & Sinus Congestion - Joint Disorders - Sleep Disorders - Acne - Thyroid Disorders - Depression - Weight loss/Gain | - Joint Disorders - Hypertension - Sleep Disorders - Hyperlipidemic Conditions - Diabetes - Thyroid Disorders - Nasal & Sinus Congestion - Eye Conditions - GERD - Low Back Pain | - Breast Cancer - Prostate Cancer - Colon Cancer - Lung Cancer - Ovarian Cancer - Chronic Lymphoid - Stomach Cancer - Non-Hodgkin’s - Bone cancer - Melanoma |

**Abbreviations:** COPD: chronic obstructive pulmonary disease; URTI: upper respiratory tract infections, GERD: Gastroesophageal reflux disorder.
